# Increasing the efficiency of mechanical ventilators during pandemics through additive manufacturing

**DOI:** 10.17305/bjbms.2020.5165

**Published:** 2021-04

**Authors:** Abdullatif Alwasel, Jean Zaky, Khalid Alhussaini, Bandar Alossimi, Turki Alharbi

**Affiliations:** 1 Department of Biomedical Technology, King Saud University, Riyadh, Kingdom of Saudi Arabia; 2 Department of Biomedical Engineering, King Saud Medical City, Riyadh, Kingdom of Saudi Arabia

**Keywords:** COVID-19, multiplexer, additive manufacturing

## Abstract

The COVID-19 pandemic tested medical facilities’ readiness in terms of the number of available mechanical ventilators. Most countries raced to stock up on ventilators, which created a surge in demand and short in supply. Furthermore, other means of coping with the demand were proposed, such as using additive manufacturing. The purpose of this paper was to test whether the addition of 3D-printed splitters would help deliver required tidal volume to each patient, while supporting four patients on a single ventilator for 24 hours on pressure mode at 25-cm H_2_O, and to determine whether a fifth patient can be ventilated. The ventilation of four human lungs was simulated using 3D printed parts, a single ventilator, four test-lungs, and standard tubing. Peak pressure, positive end-expiratory pressure, total tidal volume, individual tidal volume, total minute volume, and individual tidal volume data were collected. Usage of a 3D printed small size splitter enabled a 26% increase in individual tidal volume compared to standard tubing and a series of two-way splitters. The ventilator was able to supply the required pressure and tidal volume for 24 hours. A single ventilator with a four-way splitter can ventilate four patients experiencing respiratory failure for at least 24 hours without interruption. The equipment cannot sustain ventilating a fifth patient owing to minute volume limitation. This study expands on an earlier study that tested similar circuitry and reveals that the desired individual tidal volume is achieved. However, further research is required to provide the monitoring ability of individual patient parameters and prevention of cross-contamination.

## INTRODUCTION

Pandemics pose a multi-level challenge to healthcare systems in terms of infrastructure readiness and demand for facilities that is higher than the supplying ability. The availability and readiness of medical equipment that can be used in surge capacity is one of the major concerns during pandemics, especially if there is a substantial dependency on medical equipment. The most recent pandemic (COVID-19) required the use of mechanical ventilators for a large number of patients simultaneously. The number of ventilators required is expected to reach several hundreds of thousands to million ventilators [1]. Although these needs are manageable in well-prepared healthcare systems, such as that of the US, they are met with infrastructure limitations in certain countries. This leads to the inability of healthcare facilities to manage COVID-19 cases. Moreover, the inadequate testing of COVID-19 creates a surge in mechanical ventilators use because patients may end up using mechanical ventilators waiting for their COVID-19 results while they are normally treated using noninvasive positive-pressure ventilation (NIPPV) [1]. While proper infrastructure planning is a priority, pandemics cannot always be predicted; thus, infrastructure planning without pandemic prediction is difficult.

The surge in patients with acute respiratory distress syndrome (ARDS) due to COVID-19 requires the preparation of emergency plans by healthcare facilities. ARDS is a heterogeneous disease that progresses through several phases from inflammation of the lung cells to tissue injury and ends up with pulmonary edema, loss of surfactant, and deposition of dead cells and debris along the alveoli after direct pulmonary or indirect extrapulmonary insults. This ARDS journey leads to a wide variety of patients’ responses in terms of lung compliance [2].

Although healthcare facilities are attempting to allocate assets to cater to the demand, these emergency plans can help decrease the danger due to the surge demand for mechanical ventilators. Responding to these surges includes stockpiling equipment or surge purchase. Stockpiling on ventilators is not a common practice among healthcare facilities due to their high cost and surge purchasing is not always possible owing to the global surge demand. This set of circumstances implies that available medical equipment must be used temporarily and only in extreme emergencies until sufficient equipment is available.

A recent study utilized additive manufacturing (3D printing) to increase the efficiency (utilization) of mechanical ventilators; thus, they could operate simultaneously for two patients [3]. It was seen that the use of a 3D printed splitter and single ventilator practitioners can effectively ventilate two patients. Moreover, prior research indicated that a single mechanical ventilator is able to ventilate up to four patients, each weighing 70 kg, using a readily available connector and standard tubing [4]. Further research tested the concept of using a single ventilator on four sheep and found that it could supply sufficient ventilation to each sheep for 12 hours [5].

One of the pillars of planning for mass casualty management is the facilitation of local solutions through national policies [6]. Since the COVID-19 pandemic is concurrently generating more patients than the locally available resources, a dearth of ventilators is a likely scenario. Thus, there is a pressing need for a rapid mitigation plan, which is both affordable and accessible. In this study, we hypothesized that the increased branching of two-way splitters to use a single device on four patients can cause an increased leak in the system. Therefore, we proposed the use of a 3D printed four-way splitter, which can enhance the individual tidal volume supplied to each patient, thereby facilitating the 24-hours operation of ventilators.

## MATERIALS AND METHODS

### Study design

The presented work is a laboratory simulated study, which is exempt from review by the institutional review board.

### Study protocol

**FIGURE 1:**
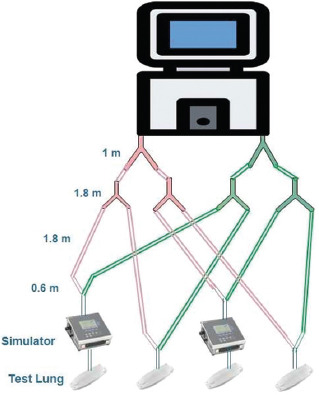
Schematic of a case wherein four patients are connected to a single ventilator using standard and readily available tubing.

**FIGURE 2:**
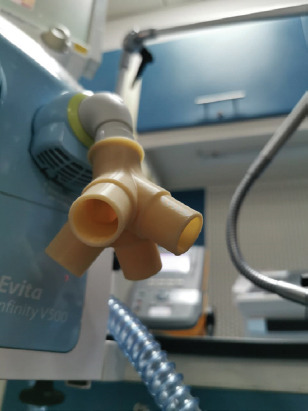
Four-way splitter placed on the inhalation branch of the ventilator.

**FIGURE 3:**
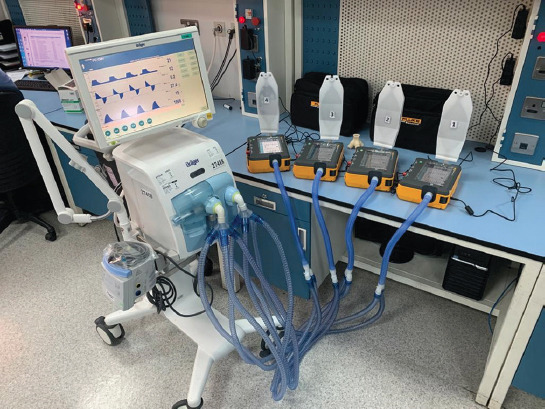
Four-way splitter implemented on a V500 ventilator with four test lungs.

In this study, two methods of connecting four patients on a single ventilator were compared. The first one uses standard tubing with standard two-way connectors, similar to the design described in the study reported in Neyman and Irvin 2006 [4]. It is shown schematically in Figure 1. The second method involves the use of a 3D printed four-way splitter, as shown in Figure 2. It comprises four sets of standard 22-mm ventilator tubing, each connected to two outlets. One is the patient inlet (inhalation branch) and the other is the patient outlet (exhalation branch), as shown in Figure 3. The parts were printed using a Zortrax^®^ M200 with a Z-ABS filament and a Form 2 Stereolithography (SLA( resin-based printer from Formlabs^®^. Each inlet branch of the ventilator tubing sets was connected to a test lung (Puritan-Bennett) and then to the outlet set of tubes returning to the ventilator. Each of the test lung branches was connected to a fluke gas analyzer to measure the individual tidal volume. A test time of 24 hours was selected for each connection method because it was assumed that overheating of the motor would limit the device, and a 24-hours period was deemed sufficient time to test its limits.

The pressure control mode was offset to 25 cm H_2_O, positive end-expiratory pressure (PEEP) was set to 5-cm H_2_O to match at least 50% of anticipated ARDS mild cases [7], and respiratory rate (R.R.) was maintained at 15 bpm.

### Measurements

Each circuitry worked continuously for 24 hours; readings from the device were monitored for any irregular behavior using the device native alarm system. Every 4 hours, each sample was recorded for reporting purposes. Regular test-lungs check observations were performed for issues such as synchronization and compliance. Test leaks were monitored by the device and recorded whenever they exceeded the permissible limit set by the device manufacturer.

## RESULTS

There were no signs of technical difficulty when using either of the methods to run a single ventilator on four simulated lungs. An attempt to connect a fifth test lung to the circuitry resulted in an alarm, indicating that the permissible limit of the minute volume of 41 L had been exceeded. No leaks beyond the permissible limit were recorded. All the readings are summarized in Table 1.

**TABLE 1:**
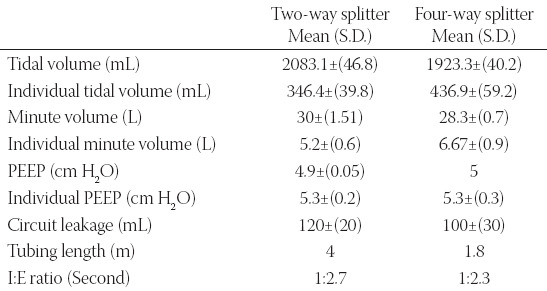
Average readings from a single ventilator connected to four patients using a two-way and a four-way splitter

## DISCUSSION

Two circuitry schemes were tested using a single ventilator on four test lungs, which served as a simulation for four patients. Two types of printers offered similar values in terms of the data presented in Table 1. However, after multiple attempts, it was found that the splitter printed using the resin-based material offered superior fitting. Moreover, the outcome suggests that a 3D printed four-way splitter can enhance the individual tidal volume supplied to each lung by 26%. The individual tidal volume delivered to each test lung using the four-ways splitter was an average of 436.9 L as compared to that using a series of two-way splitters, which was 346.4 L. This indicates that using a 3D printed four-way splitter can enhance the volume delivered to the lungs by decreasing the leak and length of tubing. Therefore, it is observed that the machine displays a superior ­performance and displays more potential endurance to operate at desired levels for as long as needed. Furthermore, the overall tidal volume of the ventilator at the source exhibits a 7% decrease using the four-way splitter compared to the two-way splitters.

These values suggest that the 3D printed four-way splitter is an efficient solution while using a single ventilator for four patients. Furthermore, the efficiency in operating the equipment could result in its long-term performance until proper equipment is acquired. This work is a laboratory simulation conducted on test lungs and needs to be verified using animal testing. This would be the next step to adhere to the cautionary measures raised by many healthcare associations [8,9]. The current study is intended for emergency use only in cases where resources are and will not be available in a reasonable time. It is assumed that all four patients are identical; thus, this is not a real-life situation. Typically treating patients with ARDS and COVID-19 will require the full control on all ventilator parameters, especially that ARDS is a heterogeneous disease and different patients may present with different lung compliances. Furthermore, same ARDS patients can have different lung compliances as the disease progresses [2]. Thus, while the assumption can be applied to patients with similar physiological characteristics, this approach is strictly not to be used except for emergencies where other solutions are not sufficient because lung-protective ventilation can never be achieved using this approach. Further testing and enhancement are required to address the need to control pressure, volume, and oxygen levels individually for each patient.

## CONCLUSION

3D printing capabilities can help in situations of surge demand for mechanical ventilators as a last resort in mass casualty management when sufficient ventilators are unavailable. The four-way splitter was able to increase the individual tidal volume by 26%, thereby facilitating the operation of the ventilator for extended periods. Further control of individual patient parameters needs to be explored in greater detail to enhance the outcome of a ventilator multiplexer, especially that ARDS patients may present with different lung compliances that require individual control abilities.
